# Association of Clinical and Economic Outcomes With Permanent Pacemaker Implantation After Transcatheter Aortic Valve Replacement

**DOI:** 10.1001/jamanetworkopen.2018.0088

**Published:** 2018-05-25

**Authors:** Talal Aljabbary, Feng Qiu, Shannon Masih, Jiming Fang, Gabby Elbaz-Greener, Peter C. Austin, Josep Rodés-Cabau, Dennis T. Ko, Sheldon Singh, Harindra C. Wijeysundera

**Affiliations:** 1 Institute for Clinical Evaluation Sciences, Toronto, Ontario, Canada; 2 Schulich Heart Centre, Sunnybrook Health Sciences Centre, University of Toronto, Toronto, Ontario, Canada; 3 Institute for Health Policy, Management and Evaluation, University of Toronto, Toronto, Ontario, Canada; 4 Quebec Heart and Lung Institute, Laval University, Quebec City, Quebec, Canada

## Abstract

**Importance:**

The literature is inconsistent regarding the impact of permanent pacemaker implantation after transcatheter aortic valve replacement.

**Objective:**

To evaluate clinical and economic outcomes in patients who required permanent pacemaker implantation during the index hospitalization after transcatheter aortic valve replacement.

**Design, Setting, and Participants:**

This retrospective, population-based cohort study using data from a multicenter registry included patients who underwent a transcatheter aortic valve replacement procedure from April 1, 2010, to March 31, 2015, in Ontario, Canada, with follow-up to March 31, 2017. Patients who had a previously implanted permanent pacemaker or who died during the index hospitalization were excluded. Inverse probability of treatment weighting using the propensity score was used to adjust for baseline differences between the pacemaker and nonpacemaker groups.

**Exposures:**

Patients received a permanent pacemaker during the index hospitalization after transcatheter aortic valve replacement.

**Main Outcomes and Measures:**

All-cause mortality, readmission, readmission for heart failure, emergency department visits, and cumulative 1-year health care costs.

**Results:**

The study cohort consisted of 1263 patients (mean [SD] age, 82.3 [7.2] years; 595 [47.1%] female; 137 [10.8%] rural), of whom 186 (14.7%) required permanent pacemaker insertion during the index hospitalization after transcatheter aortic valve replacement. Mean follow-up was 990 days. After propensity score weighting, over the entire follow-up period, pacemaker implantation was associated with significantly higher all-cause mortality (43.9% vs 31.7%; hazard ratio [HR], 1.40; 95% CI, 1.01-1.94; *P* = .04), all-cause readmission (80.9% vs 70.6%; HR, 1.28; 95% CI, 1.15-1.43; *P* < .001), and emergency department visits (95.5% vs 87.3%; HR, 1.28; 95% CI, 1.08-1.52; *P* = .004). Pacemaker implantation was also associated with significantly greater readmission for heart failure (33.9% vs 19.1%; HR, 1.90; 95% CI, 1.53-2.36; *P* < .001). There were no statistically significant differences between groups in adjusted cumulative health care costs 1 year after discharge.

**Conclusions and Relevance:**

New permanent pacemaker implantation after transcatheter aortic valve replacement was associated with significantly greater morbidity and mortality at long-term follow-up. However, this did not translate to a difference in cumulative health care costs after hospital discharge.

## Key Points

**Question:**

What are the clinical and economic outcomes in patients who required permanent pacemaker implantation after transcatheter aortic valve replacement**?**

**Findings:**

In this population-based cohort study of 1263 patients who underwent transcatheter aortic valve replacement, implantation of a new permanent pacemaker was associated with significantly greater all-cause mortality, all-cause readmission, and all-cause emergency department visits. However, this did not translate to a statistically significant difference in cumulative postdischarge health care costs.

**Meaning:**

The need for permanent pacemaker implantation after transcatheter aortic valve replacement is a complication associated with worse survival as well as increased risk of hospitalization.

## Teaser

This cohort study uses registry data to examine associations between permanent pacemaker implantation during hospitalization for transcatheter aortic valve replacement (TAVR) and mortality, hospital readmission, emergency department visits, and health care costs.

## Introduction

Transcatheter aortic valve replacement (TAVR) has become the treatment of choice for patients with severe aortic stenosis who are at high surgical risk or otherwise unsuited for an operation, with new evidence suggesting TAVR is a reasonable alternative in patients at intermediate risk.^1,2,3,4^ However, periprocedural complications remain a concern, in particular TAVR-related conduction disturbances and the need for permanent pacemaker (PPM) implantation.

The incidence of new PPM implantation following TAVR differs between types of prostheses used, with a pooled estimate of 13% in a recent meta-analysis.^5,6^ Long-term right ventricular apical pacing has been associated with mortality and rehospitalization for heart failure in patients with left ventricular dysfunction.^7,8,9,10^ Although the predictors for requiring a PPM after TAVR are well described,^11,12,13,14,15,16^ there is inconsistency regarding clinical consequences; most studies suggest neutral effects, with a few showing negative or even positive outcomes after PPM implantation.^17,18,19,20,21,22,23,24,25^ In addition, there is a paucity of data on the impact of PPM implantation on health care resource use or costs.

Accordingly, the objective of our study was to address these gaps in knowledge by comparing patients who required a PPM during the index hospitalization after TAVR with those who did not using a contemporary population-based TAVR cohort.

## Methods

This study was approved by the institutional research board at Sunnybrook Health Sciences Center, Toronto, Ontario, Canada. Our study was conducted in Canada’s largest province, Ontario, in which approximately 13.6 million residents receive publicly funded universal medical coverage provided by a single third-party payer, the Ontario Ministry of Health and Long-Term Care. The use of anonymized administrative data without patient consent at the Institute for Clinical Evaluative Sciences is allowed in Ontario based on provincial privacy legislation. The Strengthening the Reporting of Observational Studies in Epidemiology (STROBE) reporting guideline was followed.

### Data Sources

Data were obtained from the TAVR registry at CorHealth Ontario, which represents a network of 10 hospitals that performed TAVR procedures during the study period. Its accuracy has been validated by retrospective medical record review.^26,27^ We linked data from the CorHealth TAVR registry using encoded patient identifiers to population-level administrative databases housed at the Institute for Clinical Evaluative Sciences (eAppendix in the Supplement).

### Study Population and Design

This was a retrospective cohort study in which all patients who underwent a TAVR procedure from April 1, 2010, to March 31, 2015, were identified through the CorHealth TAVR registry. We included all patients who were discharged alive from the hospital following TAVR. The patients were categorized into either the PPM group or the non-PPM group based on whether they had a PPM implanted during the index TAVR hospitalization. Implantation of a PPM was identified by Canadian Classification of Health Interventions codes in the Canadian Institute for Health Information (CIHI) Discharge Abstract Database or Ontario Health Insurance Plan codes (eTable 1 in the Supplement); we included all types of PPM, including internal cardiac defibrillators, as well as cardiac resynchronization devices. Patients were excluded if they had a PPM prior to the TAVR hospitalization based on the same administrative codes in a 5-year lookback window or a record of a previous PPM in the CorHealth registry.

### Study Outcomes

The primary outcome was defined as all-cause mortality. Patients were followed up from the date of discharge after the index TAVR procedure until death or the date of last possible follow-up (March 31, 2017). Secondary outcomes were all-cause readmission and emergency department visits at longest follow-up and health care costs 1 year after discharge. A post hoc sensitivity analysis was conducted evaluating heart failure–specific hospital readmission, and we also examined readmissions for gastrointestinal bleeding and/or pneumonia as a falsification end point. Total aggregate 1-year health care costs per patient after the TAVR procedure were calculated and divided into 5 sectors: acute care, continuing care, outpatient care, physician costs, and prescription medication use. As previously noted, the cost of prescription medication use was included only in patients aged 65 years and older given that full coverage in Ontario is restricted to this age group. Costs associated with physician visits and laboratory tests were calculated based on the claims history in the Ontario Health Insurance Plan database.^28^ The resource intensity weights method was used to estimate the cost of hospital admission.^29,30,31,32^ This method provides a measure of the resources used during a patient’s encounter with the health care system. The resource intensity weights associated with the case mix group for each hospital admission in the CIHI Discharge Abstract Database were multiplied by the average provincial cost per weighted case for all Ontario acute and chronic care hospitals that year.^29,30,31,32^ The resource intensity weights method was also used to determine emergency department visit and same-day surgery costs found in the National Ambulatory Care Reporting System database. Costs were adjusted to 2016 Canadian dollars using the consumer price index.

### Statistical Analysis

Differences in baseline characteristics between patients in the PPM group and those in the non-PPM group were compared using the *t* test for continuous variables and the χ^2^ test for categorical variables. We used inverse probability of treatment weighting (IPTW) regression using the propensity score to account for the observational nature of our data set and, thus, potential differences in the distribution of measured confounders between the PPM and non-PPM groups.^33,34^ The propensity score was developed by fitting a logistic regression model with the treatment status (PPM vs non-PPM) as the dependent variable (see eTable 2 in the Supplement for covariates in the model). The adequacy of the propensity score was determined based on the standardized differences of baseline characteristics between the PPM and non-PPM groups after application of the stabilized propensity score inverse weights, with good balance indicated by standardized differences of less than 0.1.^35,36^ We did not include prosthesis type in the IPTW because previous studies have shown that although self-expanding TAVR prosthesis implantation is closely correlated with PPM, valve type is not a predictor of mortality.^37,38,39^ As an additional check, we confirmed that there was no difference in mortality between different prosthesis types in our cohort (hazard ratio [HR], 1.08; 95% CI, 0.85-1.37; *P* = .54). Prior research has shown that including variables associated only with treatment assignment and not with primary or secondary outcomes in the propensity score model can result in a loss of efficiency.^40,41^ Additional balance diagnostics were performed, including the generation of a kernel density plot and box plots, to assess whether the propensity score model had been adequately specified (ie, visual inspection of these plots can be performed to determine whether the distribution of continuous covariates is similar between the treatment and control groups) (eFigure in the Supplement). We then developed cause-specific Cox proportional hazards models weighted using IPTW to determine the association between PPM implantation and the HR of all-cause mortality, readmission, and emergency department visits.^42^ We used a robust sandwich variance estimator to account for the weighted nature of the sample and accounted for the competing risk of death in the readmission and emergency department visit models. A weighted Kaplan-Meier survival curve was constructed for each outcome in the PPM and non-PPM groups. As a sensitivity analysis, we repeated these models, including the development of the propensity score weights, excluding patients who received an implantable cardioverter-defibrillator or cardiac resynchronization therapy defibrillator device.

The mean and median costs for the PPM and non-PPM groups for overall health care costs and for each health care sector were calculated for a period of up to 1 year after discharge. To account for the highly skewed distribution of health care cost data and the small sample size, we used a generalized linear model with logarithmic link and gamma distribution to compare 1-year postdischarge health care costs between the PPM and non-PPM groups. A single predictor (PPM vs non-PPM) was used in the generalized linear model, which was weighted using IPTW. Bootstrapping was used to estimate 95% confidence intervals. Mean and median costs were compared between the PPM and non-PPM groups using the *t* test and Kruskal-Wallis test, respectively.

All data analyses were performed using SAS statistical software, version 9.4 (SAS Institute Inc). Two-sided *P* < .05 was considered statistically significant.

## Results

**Figure 1.**
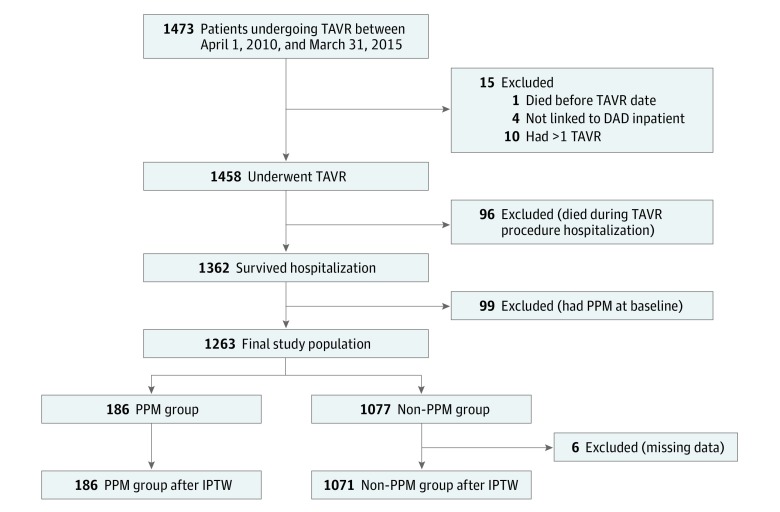
Cohort Selection All patients who underwent a transcatheter aortic valve replacement (TAVR) procedure from April 1, 2010, to March 31, 2015, were identified through the CorHealth TAVR registry. After excluding patients who had incomplete data, died during hospitalization, or had a permanent pacemaker (PPM) at baseline, patients were categorized into either the PPM group or the non-PPM group based on whether they had a PPM implanted during the index TAVR hospitalization. DAD indicates Canadian Institute for Health Information Discharge Abstract Database; IPTW, inverse probability of treatment weighting.

A total of 1473 unique patients who underwent TAVR between April 1, 2010, and March 31, 2015, were identified through the CorHealth Ontario TAVR registry. As seen in Figure 1, 15 patients were excluded because of data inconsistencies, including death before the TAVR, not being linkable to the Discharge Abstract Database due to inaccurate unique identifiers, and having more than 1 TAVR. A further 96 patients were excluded because of in-hospital death. Of the patients who died in the hospital, only 12 (12.5%) had a PPM implanted after TAVR. From the 1362 patients who survived to hospital discharge, we excluded 99 who already had a PPM at baseline. In the remaining 1263 patients, 186 (14.7%) required PPM insertion after TAVR (PPM group), and 1077 did not (non-PPM group). After IPTW, 6 patients in the non-PPM group were excluded because of missing data, resulting in a final sample size of 1071 patients in the non-PPM group.

Before IPTW, among the 1263 patients, the mean (SD) age was 82.3 (7.2) years, 595 (47.1%) were female, and 137 (10.8%) lived in rural locations (eTable 3 in the Supplement). Mean follow-up was 990 days.

After application of the propensity score weights, as seen in Table 1, the groups were well balanced, with standardized differences of less than 0.1 for all measured baseline covariates. Overall, patients were elderly (mean [SD] age, 82 [7.4] years) with a high burden of comorbidities and were more likely to be male (666 of 1257 [53.0%]). A total of 134 of 1257 participants (10.7%) were from rural locations. A small proportion of patients (86 of 1257 [6.8%]) had a diagnosis of dementia. Although the severity of dementia was not documented, we assumed it was mild, given that these patients were accepted for TAVR.

**Table 1.** Baseline Characteristics After Inverse Probability of Treatment Weighting

Characteristic	Non-PPM (n = 1071)	PPM (n = 186)	Standardized Difference
Demographics			
Age at index, y			
Mean (SD)	82.4 (7.2)	82.4 (7.7)	0.008
Median (IQR)	84 (79-87)	85 (78-88)	NA
Female, No. (%)	504 (47.1)	87 (46.7)	0.007
Rural, No. (%)	115 (10.7)	19 (10.2)	0.017
Nearest census-based neighborhood income quintile (within CMA or CA), No. (%)			
1 (Lowest income)	166 (15.5)	28 (15.2)	<0.001
2	235 (21.9)	44 (23.5)	0.037
3	232 (21.7)	45 (24.2)	0.060
4	214 (20.0)	32 (17.4)	0.067
5 (Highest income)	223 (20.8)	37 (19.7)	0.028
Clinical characteristics			
Frailty^a^	208 (19.4)	33 (17.9)	0.039
Charlson comorbidity index score			
Mean (SD)	1.9 (1.9)	1.9 (1.7)	0.004
Median (IQR)	2 (0-3)	2 (0-3)	NA
NYHA functional class at referral, No. (%)			
I	92 (8.6)	15 (8.2)	0.013
II	162 (15.2)	29 (15.7)	0.014
III	632 (59)	113 (60.9)	0.039
IV	128 (12)	19 (10.4)	0.051
STS score on the day of TAVR procedure			
Mean (SD)	8.4 (7.3)	8.9 (9.0)	0.063
Median (IQR)	6 (4-10)	6 (5-9)	NA
Left ventricular ejection fraction at referral, No. (%)			
>50%	462 (43.1)	83 (44.6)	0.029
≤50%	183 (17.1)	32 (17.2)	0.003
Missing	426 (39.8)	71 (38.2)	0.032
Aortic valve mean gradient, mm Hg			
Mean (SD)	46.1 (15.6)	46 (14.8)	0.003
Median (IQR)	45 (36-55)	45 (36-54)	NA
Cardiac conditions, No. (%)			
Myocardial infarction	137 (12.8)	21 (11.5)	0.041
Recent (<90 d) myocardial infarction hospitalization	54 (5.1)	10 (5.4)	0.013
Congestive heart failure	796 (74.3)	133 (71.5)	0.063
Recent (<90 d) heart failure hospitalization	251 (23.5)	42 (22.3)	0.027
Congestive heart failure ≥90 d	544 (50.8)	91 (49.2)	0.033
Coronary artery disease or ischemic heart disease	771 (72.0)	139 (74.5)	0.058
Prior percutaneous coronary intervention	398 (37.1)	70 (37.6)	0.001
Prior coronary artery bypass graft	282 (26.4)	48 (25.6)	0.018
Prior valve surgery or replacement	157 (14.7)	25 (13.2)	0.043
Cardiac arrhythmia or atrial arrhythmia	281 (26.2)	45 (24.3)	0.045
Noncardiac comorbidities, No. (%)			
Hypertension	1006 (93.9)	172 (92.4)	0.061
Dyslipidemia	750 (70.0)	131 (70.5)	0.011
Diabetes	508 (47.4)	85 (45.4)	0.040
Chronic obstructive pulmonary disease	381 (35.6)	63 (33.9)	0.034
Renal disease	116 (10.8)	22 (11.6)	0.024
Dementia	76 (7.1)	10 (5.4)	0.076
Cancer	72 (6.8)	12 (6.7)	0.003
Peripheral vascular disease	60 (5.6)	10 (5.3)	0.013
Cerebrovascular disease	51 (4.8)	9 (4.7)	0.003
Procedural, No. (%)			
Elective procedure	863 (80.5)	151 (81.2)	0.018
Urgent or emergent procedure	208 (19.5)	35 (18.8)	0.018
Valve-in-valve procedure	100 (9.4)	17 (9.1)	0.014
Transfemoral access route	847 (79.1)	153 (82.1)	0.076
Hemodynamic support	212 (19.8)	40 (21.6)	0.044
Postdeployment valvuloplasty	116 (10.9)	20 (10.7)	0.007

Abbreviations: CA, census agglomeration; CMA, census metropolitan area; NA, not applicable; IQR, interquartile range; NYHA, New York Heart Association; PPM, permanent pacemaker; STS, Society of Thoracic Surgeons; TAVR, transcatheter aortic valve replacement.

^a^ Frailty is based on the Johns Hopkins Adjusted Clinical Group Case mix adjustment system.

Among the PPM group, only 3 patients (1.6%) received an implantable cardioverter-defibrillator and 4 patients (2.1%) received a cardiac resynchronization therapy defibrillator device. In the non-PPM group, an additional 31 patients (2.9%) received PPM at longest follow-up following discharge date, with a mean (SD) interval from TAVR discharge to PPM insertion of 155 (125) days in this group.

**Figure 2.**
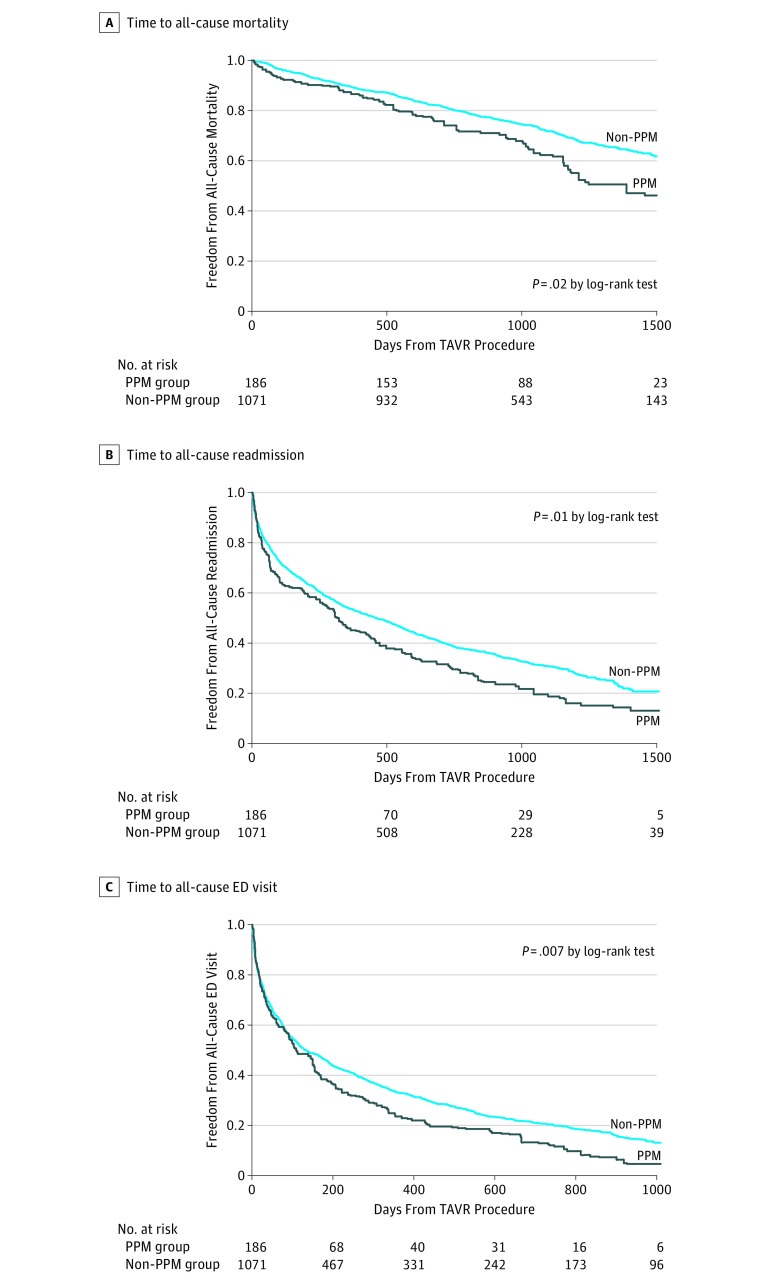
Weighted Kaplan-Meier Curves Comparing Permanent Pacemaker (PPM) vs Non-PPM Groups Over the Entire Follow-up Period New PPM after transcatheter aortic valve replacement (TAVR) was associated with a longer duration of hospitalization and significantly greater all-cause mortality (A), all-cause readmission (B), and all-cause emergency department (ED) visits (C).

Study outcomes are shown in Table 2 and Figure 2 for the sample after the application of IPTW. Over the entire follow-up period following discharge, PPM implantation was associated with significantly greater all-cause mortality (43.9% vs 31.7%; HR, 1.40; 95% CI, 1.01-1.94; *P* = .04), all-cause readmission (80.9% vs 70.6%; HR, 1.28; 95% CI, 1.15-1.43; *P* < .001), and emergency department visits (95.5% vs 87.3%; HR, 1.28; 95% CI, 1.08-1.52; *P* = .004). Similar results were seen at 1-year follow-up (Table 2). The mean (SD) length of hospital stay after TAVR was longer in the PPM group at 13.2 (12.5) days, compared with 8.8 (17.4) days in the non-PPM group (*P* = .001) (Table 3). In our sensitivity analysis of readmissions for heart failure, we found that PPM implantation was associated with significantly greater readmission for heart failure (33.9% vs 19.1%; HR, 1.90; 95% CI, 1.53-2.36; *P* < .001). In contrast, we found no difference in the proportion of admissions for the composite of gastrointestinal bleeding and pneumonia (HR, 1.27; 95% CI, 0.67-2.42; *P* = .46). When we repeated our models, excluding the 7 patients with either an implantable cardioverter-defibrillator or a cardiac resynchronization therapy defibrillator device, our findings were consistent (eTable 4 in the Supplement).

**Table 2.** Primary and Secondary Outcomes After Inverse Probability of Treatment Weighting

Outcome	Hazard Ratio (95% CI)	*P* Value
At 1 y		
All-cause mortality	1.25 (1.09-1.43)	.008
All-cause readmission	1.25 (1.12-1.39)	<.001
All-cause emergency visit	1.23 (1.04-1.45)	.01
At longest follow-up^a^		
All-cause mortality	1.40 (1.01-1.94)	.04
All-cause readmission	1.28 (1.15-1.43)	<.001
All-cause emergency visit	1.28 (1.08-1.52)	.004

^a^ Follow-up to March 31, 2017.

**Table 3.** Outcomes of Length of Stay After Inverse Probability of Treatment Weighting

Time to Discharge	Non-PPM	PPM	*P* Value
From TAVR admission to discharge, d			
Mean (SD)	11.8 (21.3)	16.3 (16.7)	.006
Median (IQR)	6 (4-11)	10 (7-17)	<.001
From TAVR to discharge, d			
Mean (SD)	8.8 (17.4)	13.2 (12.5)	.001
Median (IQR)	5 (4-8)	10 (7-14)	<.001

Abbreviations: IQR, interquartile range; PPM, permanent pacemaker; TAVR, transcatheter aortic valve replacement.

As seen in eTable 5 in the Supplement, there were no statistically significant differences in cumulative 1-year postdischarge health care costs between the PPM group and the non-PPM group (cost ratio, 1.18; 95% CI, 0.91-1.37; *P* = .28). The mean (SD) and median (interquartile range) 1-year health care costs for the PPM group after hospital discharge were $38 310 ($50 410) and $23 566 ($13 218-$45 161), respectively, while for the non-PPM group the mean and median costs were $34 254 ($42 970) and $18 108 ($10 694-$38 434). Postdischarge health care costs were predominantly driven by inpatient hospitalizations. The costs for inpatient hospitalization after the index TAVR discharge were higher in the PPM group, with a median (interquartile range) cost of $7764 ($3694-$18 059), compared with a median (interquartile range) cost of $5915 ($3263-$15 399) in the non-PPM group.

## Discussion

Using a population-level cohort of all patients who underwent TAVR in Ontario, Canada, we found that PPM implantation after TAVR was required in approximately 15% of patients. Implantation of a new PPM after TAVR was associated with a longer duration of hospitalization and significantly greater all-cause mortality, all-cause readmission, and all-cause emergency department visits at 1 year and long-term follow up.

Previous studies have shown inconsistency regarding the clinical impact of post-TAVR PPM implantation.^5^ A large series of 1556 patients undergoing TAVR by Urena and colleagues^24^ showed no association of new PPM implantation after TAVR with overall or cardiovascular mortality or repeated hospitalization after a mean follow-up of approximately 2 years. Similarly, a German registry of 1147 patients showed no association of new PPM implantation with 30-day mortality, but did not examine long-term outcomes.^43^ Mouillet et al^21^ reported that new PPM implantation among recipients of a CoreValve from the French Aortic National CoreValve and Edwards (FRANCE-2) registry was not associated with long-term all-cause or cardiovascular mortality. Other smaller series also demonstrated no association of PPM implantation with 1-year and 2-year all-cause mortality,^17,18,19,20,23^ with 1 suggesting improved survival at 1 year and 5 years.^25^

However, a substudy of the Placement of Aortic Transcatheter Valves (PARTNER) data demonstrated an association of new PPM implantation with increased length of stay and an increase in the composite of hospitalization or mortality after TAVR at 1 year.^22^ These 1-year adverse outcomes were also seen by Fadahunsi and colleagues in an analysis of 9785 patients in the US Transcatheter Valve Therapy registry.^44^ However, it is difficult to draw comparisons with the available data, given the different prostheses and periods in addition to the relatively short follow-up periods.

In contrast to most other published reports, this analysis examined a contemporary, population-based cohort that included all prosthesis types over a substantially longer follow-up period. Another potential explanation for our discordant results is that we excluded all immediate postprocedural in-hospital deaths from our analysis, in contrast to most previous studies. These early deaths would be allocated to the non-PPM group and may potentially result in an immortal time or survivorship bias. Indeed, we saw that 87.5% of in-hospital deaths did not have a PPM and would have been assigned to the non-PPM group. We have mitigated this issue by excluding those deaths and essentially doing a landmark analysis from the time of index hospital discharge. We would argue that for evaluation of mid- and long-term consequences, immediate periprocedural deaths, which are typically the result of complications, should be excluded because they would not be attributable to the presence or absence of a PPM.

Our study was not designed to elucidate the underlying mechanisms for the observation of increased morbidity and mortality with PPM. We can hypothesize as to several potential reasons. First, long-term right ventricular apical pacing has been associated with an increased rate of mortality and readmission for heart failure in patients with structural heart disease as well as an increased incidence of pacing-induced cardiomyopathy in patients without overt structural heart disease.^8,9,10,11^ Indeed, our finding of increased heart failure–related hospitalization associated with PPM implantation after TAVR is consistent with these previous findings. Second, patients with conduction disorders that are unmasked by the TAVR procedure may have more myocardial fibrosis, which may predispose these patients to heart failure and sudden cardiac death from ventricular arrhythmias.^45^ Finally, PPM implantation can lead to several life-threatening complications due to device implantation itself, such as infection or potential lead dislodgement in dependent patients, although we would expect this to be a very rare event.^46^ It is important to recognize that a similar adverse long-term impact of PPM has been reported with surgical aortic valve replacement.^47^ We believe that this finding requires further study to understand the potential causal mechanisms.

Interestingly, despite significantly higher rates of readmission and emergency department visits in the PPM group, we found that, although there were numerically higher mean and median costs among patients who received a PPM after TAVR compared with those who did not, this did not reach statistical significance. We postulate that this lack of statistical significance may be due to our study being underpowered given the wide variation associated with cost data; alternatively, it may be due to the increased mortality in the PPM group, which reduced the overall follow-up on average for patients with a PPM, and thus cumulative costs.

In previous US studies, the total 1-year follow-up cost was lower than what we reported in our study. In a cost analysis of the PARTNER A trial by Reynolds and colleagues,^48^ the total 1-year follow-up cost from hospital discharge was $24 787 for transfemoral TAVR and $18 856 for transapical TAVR.^48^ In another study by Reynolds et al,^49^ the total 1-year follow-up cost was $28 766 for TAVR with self-expanding prosthesis. As with our analysis, the major driver for 1-year cumulative costs in both studies was follow-up hospitalizations.

### Limitations

Our study must be interpreted in the context of several limitations that merit discussion. First, this was an observational study, and despite the use of sophisticated statistical methods, we cannot discount the possibility of residual confounding. For example, we did not include information on baseline conduction abnormalities or atrial fibrillation, as we did not have that information in the registry. As such, our findings should be considered hypothesis generating. That said, we evaluated subsequent readmissions for gastrointestinal bleeding and/or pneumonia as a falsification end point and found no difference. Second, a large proportion of our population (approximately 40%) had missing values for left ventricular ejection fraction, an important prognostic variable. Third, we were not able to include the indication for PPM implantation, as this was not part of our abstraction data set. Fourth, pacing dependency and right ventricular pacing burden were not systematically evaluated, nor were changes in ejection fraction on follow-up. Finally, this study focused on costs after discharge and did not include the cost of the index hospitalization. Given the longer length of stay associated with PPM implantation, we would expect a substantially higher cost for this period in patients who required a PPM. We elected to focus on postdischarge costs given that we restricted our cohort to patients who survived the index hospitalization. Nonetheless, it is crucial when contextualizing our cost data to recognize the upfront additional costs associated with a PPM.

## Conclusions

Among patients who underwent TAVR, PPM implantation after TAVR was associated with significantly higher mortality and morbidity over long-term follow-up.

## Notes

Supplement. **eAppendix.** Administrative Datasets **eFigure. **Balance Statistics **eTable 1.** Codes to Identify Device Implantation **eTable 2.** Propensity Model **eTable 3.** Baseline Characteristics Before Inverse Probability of Treatment Weighting **eTable 4.** Primary and Secondary Outcomes After Inverse Probability of Treatment Weighting Excluding ICD and CRT Patients **eTable 5.** 1-Year Health Care Costs Following Hospital Discharge
