# Supplementary material for: Association of Clinical and Economic Outcomes With Permanent Pacemaker Implantation After Transcatheter Aortic Valve Replacement
Source: JAMA Netw Open. 2018 May 25;1(1):e180088. doi: 10.1001/jamanetworkopen.2018.0088 (PMC6324315; doi:10.1001/jamanetworkopen.2018.0088)
Supplement: Supplement. — eAppendix. Administrative Datasets eFigure. Balance Statistics eTable 1. Codes to Identify Device Implantation eTable 2. Propensity Model eTable 3. Baseline Characteristics Before Inverse Probability of Treatment Weighting eTable 4. Primary and Secondary Outcomes After Inverse Probability of Treatment Weighting Excluding ICD and CRT Patients eTable 5. 1-Year Health Care Costs Following Hospital Discharge [file jamanetwopen-1-e180088-s001.pdf]

Supplementary Online Content

Aljabbary T, Qiu F, Masih S, et al. Association of clinical and economic outcomes with permanent pacemaker implantation after transcatheter aortic valve replacement. *JAMA Netw Open*. 2018;1(1):e180088. doi:10.1001/jamanetworkopen.2018.0088

eAppendix. Administrative Datasets
eFigure. Balance Statistics
eTable 1. Codes to identify device implantation
eTable 2. Propensity model
eTable 3. Baseline Characteristics Before Inverse Probability of Treatment Weighting
eTable 4. Primary and Secondary Outcomes After Inverse Probability of Treatment Weighting excluding ICD and CRT patients
eTable 5. 1-year health care costs following hospital discharge

This supplementary material has been provided by the authors to give readers additional information about their work.

© 2018 Aljabbary T et al. *JAMA Network Open*.

eAppendix: Administrative Datasets

The administrative databases used in our study included the Registered Persons Database (RPDB), which contains demographic information, and was used to ascertain mortality. The Ontario Health Insurance Plan (OHIP) claims database contains all fee-for-service billing claims made by Ontario physicians. The Canadian Institute for Health Information Discharge Abstract Database (CIHI-DAD) contains administrative and clinical data on all inpatient hospitalizations. The National Ambulatory Care Reporting System (NACRS) captures data on emergency department (ED) visits, same day surgery (SDS) procedures and visits to ambulatory clinics. The National Rehabilitation Reporting System (NRS) database holds data on all admissions to in-hospital rehabilitation beds. The Continuing Care Reporting System (CCRS) provides information on individuals receiving long-term care. The Ontario Mental Health Reporting System (OMHRS) captures administrative and clinical data on all adult mental health admissions. The Home Care Database (HCD) contains demographic and service information for individuals receiving in-home services. The Ontario Drug Benefit (ODB) Program provides data on prescription medications dispensed to individuals aged 65 and older.

© 2018 Aljabbary T et al. *JAMA Network Open*.

eFigure: Balance Statistics

a) Kernel density estimate of propensity score comparing raw (unweighted) VS weighted.

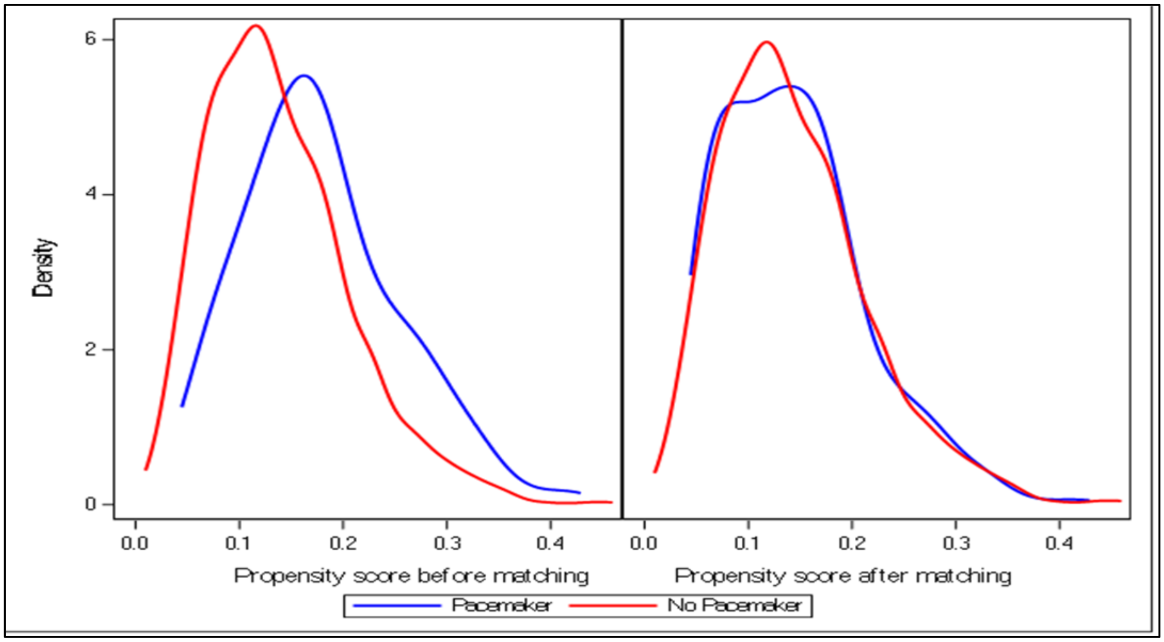

b) Box plot of propensity score comparing raw (unweighted) versus weighted.

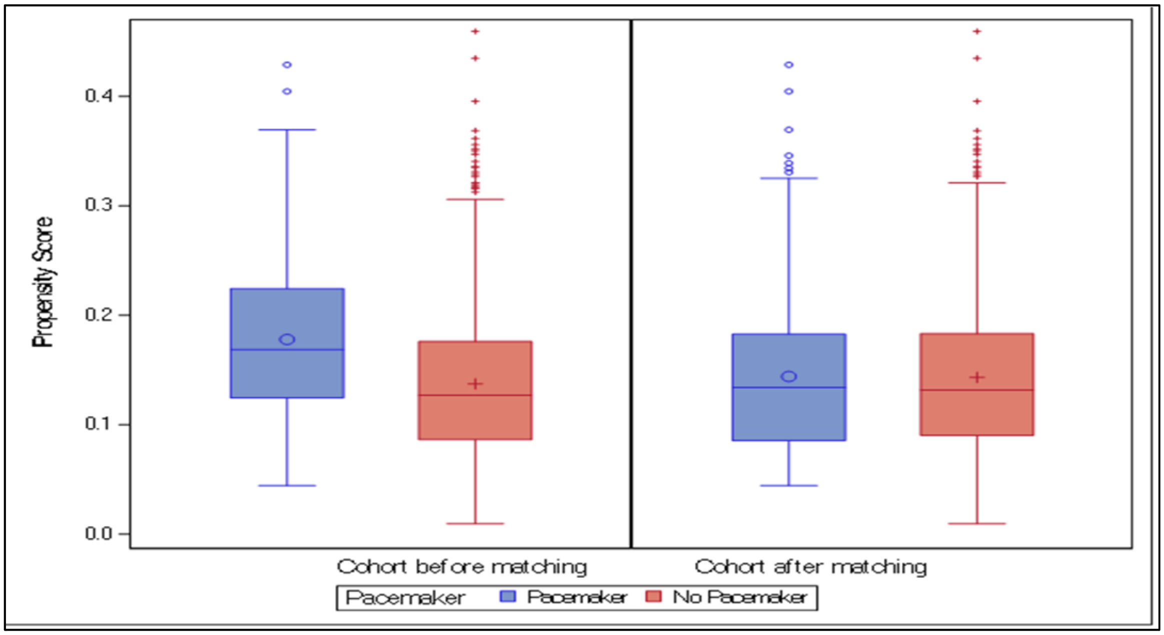

© 2018 Aljabbary T et al. *JAMA Network Open*.

eTable 1: Codes to identify device implantation

Procedure	CCI codes[a]	OHIP codes
Permanent pacemaker implantation	1HZ53GRNM, 1HZ53GRNK, 1HZ53GRNL	R752, Z444
Implantable cardioverter defibrillator (ICD)	1HZ53GRFS	R761
Cardiac resynchronization therapy pacemaker (CRT-P)	1HZ53GRFR	Z429
Cardiac resynchronization therapy defibrillator (CRT-D)	1HZ53GRFU	R761, Z429

CCI: Canadian Classification of Health Interventions; OHIP: Ontario health insurance program
[a] CCI codes are found in the Canadian Institute of Health Information (CIHI) discharge abstract database (DAD)

© 2018 Aljabbary T et al. *JAMA Network Open*.

eTable 2: Propensity model

Effect	Odds Ratio Estimate	Lower CL	Upper CL
**Demographics**			
Age	0.10	0.97	1.02
Female	0.53	0.37	0.76
Rural	1.18	0.71	1.94
Income Quintile 2 vs 1	0.91	0.54	1.55
Income Quintile 3 vs 1	1.04	0.62	1.75
Income Quintile 4 vs 1	0.76	0.44	1.31
Income Quintile 5 vs 1	0.72	0.42	1.26
**Clinical Characteristics**			
Frailty	0.73	0.44	1.21
Charlson Score	0.91	0.77	1.08
NYHA Class II vs Class I	3.86	1.60	9.61
NYHA Class III vs Class I	2.67	1.12	6.34
NYHA Class IV vs Class I	3.07	1.15	8.21
Missing NYHA class vs Class I	5.03	1.79	14.11
LVEF >50% vs LVEF<=50	1.46	0.89	2.39
Missing LV function vs LVEF<=50	0.99	0.59	1.66
**Cardiac conditions**			
MI	1.32	0.76	2.29
CAD	0.94	0.61	1.44
Recent CHF vs No CHF	1.39	0.79	2.43
CHF not Recent vs No CHF	1.25	0.83	1.89
Cardiac Arrhythmia/Atrial Arrhythmia	1.02	0.69	1.51
Prior PCI	1.26	0.87	1.81
Prior CABG	0.98	0.65	1.46
Prior valve surgery/replacement	0.97	0.50	1.90
**Non- Cardiac co-morbidities**			
Diabetes	1.23	0.83	1.81
Hypertension	1.12	0.55	2.31
Dyslipidemia	1.14	0.78	1.66
PVD	2.10	1.09	4.04
Cerebrovascular Disease (CVD)	0.68	0.27	1.71
COPD	1.17	0.83	1.64
Dementia	0.73	0.33	1.62
Cancer	0.86	0.39	1.88
Renal disease	0.95	0.49	1.83
**Procedural characteristics**			

© 2018 Aljabbary T et al. *JAMA Network Open*.

Effect (continued)	Odds Ratio Estimate	Lower CL	Upper CL
Hemodynamic support	0.80	0.52	1.25
Valve in valve	0.54	0.22	1.29
Access Route			
Trans-femoral	1.13	0.74	1.71
Procedure urgency			
Urgent/Emergent vs Elective	0.89	0.56	1.40

CAD= coronary artery disease; CABG= coronary artery bypass graft; COPD= chronic obstructive pulmonary disease; CHF= congestive heart failure; CVD= Cerebrovascular Disease; MI= myocardial infraction; NYHA= New-York Heart Association; PCI=percutaneous coronary intervention; PPM= Permanent pacemaker; PVD= Peripheral Vascular Disease; LV=left ventricle; LVEF= left ventricle ejection fraction

© 2018 Aljabbary T et al. *JAMA Network Open*.

eTable 3: Baseline Characteristics Before Inverse Probability of Treatment Weighting

Patient Characteristics	Non- PPM group	PPM group	TOTAL
	N= 1,077	N= 186	N= 1,263
**Demographics**			
Age at index			
Mean (STD)	82.34 ± 7.20	82.10 ± 7.35	82.31 ± 7.22
Median (IQ)	84 (79-87)	84 (77-88)	84 (78-87)
Female, No. (%)	530 (49.2%)	65 (34.9%)	595 (47.1%)
Rural, No. (%)			
N	963 (89.4%)	163 (87.6%)	1,126 (89.2%)
Y	114 (10.6%)	23 (12.4%)	137 (10.8%)
Nearest Census Based Neighbourhood Income Quintile (within CMA/CA), No. (%)			
1 (lowest)	165 (15.3%)	31 (16.7%)	196 (15.5%)
2	235 (21.8%)	41 (22.0%)	276 (21.9%)
3	226 (21.0%)	45 (24.2%)	271 (21.5%)
4	218 (20.2%)	34 (18.3%)	252 (20.0%)
5 (highest)	227 (21.1%)	35 (18.8%)	262 (20.7%)
**Clinical Characteristics**			
Frailty, No. (%)	221 (20.5%)	26 (14.0%)	247 (19.6%)
Charlson score			
Mean (STD)	1.93 ± 1.91	1.88 ± 1.69	1.92 ± 1.88
Median (IQ)	2 (0-3)	2 (0-3)	2 (0-3)
NYHA functional class at referral, No. (%)			
Class I	104 (9.7%)	6 (3.2%)	110 (8.7%)
Class II	151 (14.0%)	39 (21.0%)	190 (15.0%)
Class III	639 (59.3%)	105 (56.5%)	744 (58.9%)
Class IV	131 (12.2%)	21 (11.3%)	152 (12.0%)
Missing	52 (4.8%)	15 (8.1%)	67 (5.3%)
STS score on The Day of TAVR Procedure			
Mean (STD)	8.48 ± 7.34	9.71 ± 9.93	8.64 ± 7.71
Median (IQ)	6 (4-10)	6 (5-9)	6 (4-10)
LV Function at referral, No. (%)			
LVEF >50%	455 (42.2%)	88 (47.3%)	543 (43.0%)
LVEF 31-50%	144 (13.4%)	24 (12.9%)	168 (13.3%)
LVEF 21-30%	37 (3.4%)	<=5	41 (3.2%)
LVEF <=20%	<=5	<=5	7 (0.6%)
Missing	436 (40.5%)	68 (36.6%)	504 (39.9%)
AV Mean Gradient			
Mean (STD)	46.12 ± 15.68	44.68 ± 14.49	45.89 ± 15.50

© 2018 Aljabbary T et al. *JAMA Network Open*.

Patient Characteristics (continued)	Non- PPM group	PPM group	TOTAL
	N= 1,077	N= 186	N= 1,263
**Cardiac conditions**			
Median (IQ)	45 (36-55)	44 (35-52)	45 (36-55)
Myocardial infarction (MI), No. (%)	135 (12.5%)	27 (14.5%)	162 (12.8%)
Recent (<90 days) MI Hospitalization, No. (%)	54 (5.0%)	14 (7.5%)	68 (5.4%)
Heart failure (CHF), No. (%)	798 (74.1%)	142 (76.3%)	940 (74.4%)
Recent (<90 days) Heart Failure Hospitalization, No. (%)	254 (23.6%)	43 (23.1%)	297 (23.5%)
CAD/Ischemic Heart Disease, No. (%)	770 (71.5%)	140 (75.3%)	910 (72.1%)
Priori PCI, No. (%)	390 (36.2%)	79 (42.5%)	469 (37.1%)
Prior CABG, No. (%)	276 (25.6%)	55 (29.6%)	331 (26.2%)
Prior valve surgery/replacement, No. (%)	164 (15.2%)	21 (11.3%)	185 (14.6%)
Cardiac Arrhythmia/Atrial Arrhythmia, No. (%)	285 (26.5%)	47 (25.3%)	332 (26.3%)
**Non- Cardiac co-morbidities**			
Diabetes, No. (%)	505 (46.9%)	95(51.1%)	600 (47.5%)
Hypertension, No. (%)	1,011 (93.9%)	176 (94.6%)	1,187 (94.0%)
Dyslipidemia, No. (%)	747 (69.4%)	138 (74.2%)	885 (70.1%)
Peripheral Vascular Disease (PVD), No. (%)	55 (5.1%)	16 (8.6%)	71 (5.6%)
Cerebrovascular Disease (CVD), No. (%)	54 (5.0%)	6 (3.2%)	60 (4.8%)
COPD, No. (%)	379 (35.2%)	73 (39.2%)	452 (35.8%)
Dementia, No. (%)	81 (7.5%)	9 (4.8%)	90 (7.1%)
Cancer, No. (%)	75 (7.0%)	10 (5.4%)	85 (6.7%)
Dialysis, No. (%)	38 (3.5%)	<=5	42 (3.3%)
Renal Disease, No. (%)	118 (11.0%)	18 (9.7%)	136 (10.8%)
Liver Disease, No. (%)	14 (1.3%)	0 (0.0%)	14 (1.1%)
Interstitial Lung Disease, No. (%)	12 (1.1%)	<=5	16 (1.3%)
**Procedural characteristics**			
Procedure urgency, No. (%)			
Elective	863 (80.1%)	153 (82.3%)	1,016 (80.4%)
Urgent/Emergent	214 (19.9%)	33 (17.7%)	247 (19.6%)
Valve in Valve, No. (%)	107 (9.9%)	11 (5.9%)	118 (9.3%)
Access Route, No. (%)			
Non-trans femoral	227 (21.1%)	36 (19.4%)	263 (20.8%)
Trans-femoral	847 (78.6%)	150 (80.6%)	997 (78.9%)
Type of valve, No. (%)			
Self -expandable	331 (30.7%)	32 (17.2%)	363 (28.7%)
Balloon-expandable	252 (23.4%)	76 (40.9%)	328 (26.0%)
Other	42 (3.9%)	<=5	46 (3.6%)
Missing	452 (42.0%)	74 (39.8%)	526 (41.6%)
Hemodynamic Support, No. (%)	212 (19.7%)	37 (19.9%)	249 (19.7%)
Post-deployment valvuloplasty, No. (%)	117 (10.9%)	17 (9.1%)	134 (10.6%)

© 2018 Aljabbary T et al. *JAMA Network Open*.

Anaesthesia type, No. (%)			
General	1,044 (96.9%)	183 (98.4%)	1,227 (97.1%)

AV= aortic valve; CA= census agglomeration; CABG= coronary artery bypass graft; CAD= coronary artery disease; CHF= congestive heart failure; CMA= census metropolitan area; COPD= chronic obstructive pulmonary disease; CVD= Cerebrovascular Disease; LV=left ventricle; LVEF= left ventricle ejection fraction; MI= myocardial infraction; NYHA= New-York Heart Association; PCI=percutaneous coronary intervention; PPM= Permanent pacemaker; PVD= Peripheral Vascular Disease; SD= Standard deviation; STS= The Society of Thoracic Surgeons; TAVR= Trans-catheter aortic valve replacement

© 2018 Aljabbary T et al. *JAMA Network Open*.

eTable 4: Primary and Secondary Outcomes After Inverse Probability of Treatment Weighting excluding ICD and CRT patients.

Outcomes within 1 year	Non-PPM group	PPM-group	Hazard ratio (95%)	*P*-value
All-cause mortality	111 (10.3%)	22 (12.4%)	1.235(1.061-1.437)	0.006
All-cause re-hospitalization	494 (46.1%)	97 (54.4%)	1.244(1.098-1.41)	0.0006
All-cause emergency visit	936 (87.4%)	171 (95.4%)	1.229(1.038-1.455)	0.016
**Outcomes at longest follow up [a]**				
All-cause mortality	340 (31.7%)	79 (44%)	1.412(1.013-1.969)	0.041
All-cause re-hospitalization	757 (70.7%)	145 (81.1%)	1.297(1.156-1.456)	<.0001
All-cause emergency visit	936 (87.4%)	171 (95.4%)	1.276(1.073-1.518)	0.005

[a] Follow up to March 31st, 2017

CRT= Cardiac resynchronization therapy; ICD= Implantable cardioverter defibrillator; PPM= Permanent pacemaker.

© 2018 Aljabbary T et al. *JAMA Network Open*.

eTable 5: 1-year health care costs following hospital discharge.

Variables	Non-PPM	PPM	Standardized difference
**Total cost AFTER index date, including index date**			
Mean (STD)	34,254 (42,970)	38,310 (50,410)	0.09
Median (IQ)	18,108 (10,694-38,434)	23,566 (13,218-45,161)	
**Inpatient (DAD) cost AFTER index date, including index date**			
Mean (STD)	14,504 (25,611)	17,191.8 (33,259)	0.09
Median (IQ)	5,915 (3,263-15,399)	7,764 (3,694-18,059)	
**Same Day Surgery (SDS) cost AFTER index date, including index date**			
Mean (STD)	307 (995)	245 (691)	0.07
Median (IQ)	0 (0-0)	0 (0-0)	
**NACRS ED cost AFTER index date, including index date**			
Mean (STD)	842 (1053)	912 (1184)	0.06
Median (IQ)	519 (0-1275)	557 (137-1097)	
**Total OHIP Cost AFTER index date including index date**			
Mean (STD)	4,438 (3,784)	4,614 (4,276)	0.044
Median (IQ)	3,202 (1,922-5,457)	3,800 (2,145-5,651)	
**Total OHIP physician Cost AFTER index date including index date**			
Mean (STD)	4,103 (3,726)	4,286 (4,222)	0.046
Median (IQ)	2,808 (1,671-5,074)	3249 (1,904-5,317)	
**OHIP non-physician cost AFTER index date, including index date**			
Mean (STD)	56 (162)	58 (174)	0.017
Median (IQ)	26 (0-49)	0 (0-47)	
**OHIP lab cost AFTER index date, including index date**			
Mean (STD)	278 (279)	268 (268)	0.038
Median (IQ)	203 (86-382)	212 (65-389)	
**LTC cost (total) AFTER index date, including index date**			
Mean (STD)	859 (4869)	1164 (5248)	0.06
Median (IQ)	0 (0-0)	0 (0-0)	
**CCC cost AFTER index date, including index date**			
Mean (STD)	1854 (12290)	2131 (14223)	0.021
Median (IQ)	0 (0-0)	0 (0-0)	

© 2018 Aljabbary T et al. *JAMA Network Open*.

Variables (continued)	Non-PPM	PPM	Standardized difference
**Home Care Services cost AFTER index date, including index date**			
Mean (STD)	2,489 (5,035)	2,862 (4,966)	0.08
Median (IQ)	805 (0-2,487)	1,030 (0-3,146)	
**Rehab (NRS) cost AFTER index date, including index date**			
Mean (STD)	2,213 (7,866)	2,937 (7316)	0.10
Median (IQ)	0 (0-0)	0 (0-3190)	
**ODB drug cost (all ages) AFTER index date, including index date**			
Mean (STD)	2,917 (5,491)	2,424 (3,082)	0.11
Median (IQ)	1,876 (1,011-3,222)	1,949 (945-2,883)	
**Hospital outpatient clinic cost AFTER index date, including index date**			
Mean (STD)	1,631 (1,523)	2,014 (1,839)	0.23
Median (IQ)	1,260 (639-2,243)	1,571 (650-2591)	
**Physician capitation cost AFTER index date, including index date**			
Mean (STD)	297 (226)	301 (238)	0.020
Median (IQ)	352 (67-486)	349 (64-513)	

Index date= TAVR discharge date
CCC= complex continuing care; DAD= Discharge Abstract Database; LTC= Long Term Care; ODB= Ontario Drug Benefit; OHIP= Ontario Health Insurance Plan; NACRS= The National Ambulatory Care Reporting System; NRS= The National Rehabilitation Reporting System

© 2018 Aljabbary T et al. *JAMA Network Open*.
